# A chromosome-level reference genome for the critically endangered Southern Corroboree frog ( *Pseudophryne corroboree*)

**DOI:** 10.12688/wellcomeopenres.23820.1

**Published:** 2025-04-30

**Authors:** Tiffany A. Kosch, Lee Berger, Andrew J. Crawford, Deon Gilbert, Damian Goodall, David Hunter, Michael McFadden, Linelle Abueg, Giulio Formenti, Joanna Collins, Tatiana Tilley, Nivesh Jain, Brian O'Toole, Conor Whelan, Ira Cooke, Matthieu Muffato, Bethan Yates, Olivier Fedrigo, Jennifer Balacco, Erich D. Jarvis, Lee F. Skerratt

**Affiliations:** 1 Melbourne Veterinary School, Faculty of Science, The University of Melbourne, Melbourne, Victoria, Australia; 2 Departamento de Ciencias Biológicas, Universidad de Los Andes, Bogotá, Colombia; 3 Wildlife Conservation and Science, Zoos Victoria, Parkville, Victoria, Australia; 4 NSW Department of Climate Change, Energy, Environment, and Water, Parramatta, NSW, Australia; 5 Taronga Institute of Science and Learning, Taronga Conservation Society Australia, Mosman, New South Wales, Australia; 6 The Vertebrate Genome Laboratory, The Rockefeller University, New York, New York, USA; 7 Wellcome Sanger Institute, Hinxton, England, UK; 8 Centre for Tropical Bioinformatics and Molecular Biology, James Cook University, Townsville City, Queensland, Australia; 9 Colossal Bioscienes, Dallas, Texas, USA

**Keywords:** Genome assembly, reference genome, Anura, Critically Endangered, Myobatrachidae, conservation breeding

## Abstract

The Southern Corroboree frog ( *Pseudophryne corroboree*; Anura; Myobatrachidae) is a Critically Endangered amphibian, according to the IUCN, and is endemic to the Snowy Mountains region of Kosciuszko National Park in New South Wales, Australia. This species has been driven to functional extinction by the introduction of the fungal disease, chytridiomycosis. Here we provide the first reference genome for *P. corroboree*. Using PacBio HiFi sequencing, Arima Hi-C, and Bionano optical mapping, we produced a chromosome-level genome assembly. Additionally, we generated a reference transcriptome based on multiple tissues from both male and female individuals to support genome annotation. The resulting genome spans 8.87 Gb across 12 chromosomes, with a contig N50 of 6.8 Mb. This research provides a phased, annotated genome assembly along with transcriptomic resources to facilitate future conservation genomic studies of *P. corroboree*. Furthermore, the genome offers an invaluable resource for taxonomic and evolutionary research, particularly given the nearest available chromosome-level reference genome is from *Mixophyes fleayi*, a species that last shared a common ancestor with *P. corroboree* 80 million years ago.

## Species taxonomy

Eukaryota; Opisthokonta; Metazoa; Eumetazoa; Bilateria; Deuterostomia; Chordata; Craniata; Vertebrata; Gnathostomata; Teleostomi; Euteleostomi; Sarcopterygii; Dipnotetrapodomorpha; Tetrapoda; Amphibia; Batrachia; Anura; Neobatrachia; Myobatrachoidea; Myobatrachidae; Myobatrachinae; Pseudophryne *Pseudophryne corroboree* Moore, 1953 (NCBI:txid495146).

## Background

The genome of a Southern Corroboree frog ( *Pseudophryne corroboree*), referred to as “Gyack” by Australian Indigenous people ^1^, was sequenced as part of the Vertebrate Genomes Project (VGP), a collaborative effort aiming to produce high-quality reference genomes of all named vertebrate species ^2, 3^. Here we present a chromosome-level complete genome assembly for *P. corroboree*, from a male captive-bred specimen (BioSample SAMN32631236) provided by Melbourne Zoo (Victoria, Australia).

The Southern Corroboree frog is one of Australia’s most threatened species of amphibians ^4^. Driven to functional extinction in the wild after the introduction of the fungal pathogen, *Batrachochytrium dendrobatidis*, into Australia in the late 1970s, this species is now reliant on *ex situ* conservation breeding for its continued existence ^5, 6^.

*Pseudophryne corroboree* is a sub-alpine frog restricted to sphagnum peat bogs from 1300 and 1760 meters in elevation in Kosciuszko National Park in the Snowy Mountains region of New South Wales (NSW), Australia ^7^. The female lays terrestrial eggs in mid- to late summer that are attended by the male throughout development ^8, 9^. Eggs hatch into tadpoles after flooding by winter rains ^8^. *Pseudophryne corroboree* have bright yellow and black coloration. Unlike many other poisonous frogs (e.g., Dendrobatidae, Mantellidae), they are able to synthesize toxins in addition to obtaining them from their diets ^10^. The sister species, *P. pengilleyi*, has declined more slowly ^4, 11^, which may be due to greater resistance to chytridiomycosis in this species.

Various efforts are underway to restore *P. corroboree* to the wild including conservation breeding and reintroduction, *in situ* enclosures, conservation genomics analysis, and targeted genetic intervention ^6, 12– 15^. Here we report a high-quality reference genome and transcriptome to serve as a key resource for these efforts by facilitating informed genetic management of the species and efforts to increase resistance to the chytrid pathogen ^12, 16^.

## Methods

### Sample acquisition

An adult captive-bred male *P. corroboree* (Zoo Record ID: B50597), obtained from the conservation breeding program at Melbourne Zoo (Victoria, Australia), was used to generate the reference genome. Two additional captive animals: 1 male (B40300; the sire of the focal animal, wild-collected from unknown location in 2007) and 1 female (B40182; wild-collected from Upper Jagumba, NSW in 2007) were also sequenced using short reads. All frogs were humanely euthanized at the University of Melbourne (Victoria, Australia) using a buffered solution of 0.2% tricaine mesylate (MS-222) and decapitation (University of Melbourne Animal Ethics permit #10267). Using sterile techniques, multiple tissues including heart, liver, kidney, muscle, gonads, brain, and whole body were collected from each animal and flash frozen in liquid nitrogen. The tissues were immediately transferred to -80°C until they were sent for genome sequencing. Tissues were shipped on dry ice to the Vertebrate Genomics Laboratory (Rockefeller University, New York, USA) for nucleotide extraction and sequencing (Australian wildlife trade export permit #PWS2020-AU-001530).

### Nucleic acid extraction and sequencing

For PacBio HiFi sequencing, high molecular weight DNA (HMW DNA) was extracted from a flash frozen kidney using the MagAttract HMW DNA Kit (Qiagen 67563). The tissue was stored at -80°C and kept on dry ice until homogenized with the Qiagen TissueRuptor II (Cat. No. 9002755). The DNA was quantified with the Qubit 3 fluorometer (Invitrogen Qubit dsDNA Broad Range Assay cat no. Q32850) and fragment size was assessed with the Agilent Fragment Analyzer.

After isolation, DNA was sheared using the Megaruptor 3 (Diagenode, Denville, NJ, USA) to attain a 15–20 Kb PacBio library insert size. The PacBio HiFi library was prepared with the SMRTbell Express Template Prep Kit 2.0 (PN 100-938-900) following the manufacturer’s procedure (PN 101-853-100 Version 03) and then size-selected with Pippin HT (Sage Science, Beverly, MA, USA). The PacBio library was sequenced on a Sequel IIe instrument with 8M SMRT cells and Sequencing Plate 2.0 (PN 101-820-200).

For Hi-C sequencing, libraries were prepared from liver using the Arima-HiC 2.0 kit (Arima Genomics, Carlsbad, CA, USA) following the manufacturer’s protocol. The library was then sequenced with the Illumina NovaSeq 6000 platform with 2×150 bp read length at Psomagen, Inc. (Rockville, MD, USA).

For Bionano optical mapping, HMW DNA was extracted from the kidney with the Circulomics Nanobind Tissue Big DNA Kit and fragment size was evaluated with a pulsed field gel electrophoresis (Pippin Pulse, SAGE Science, Beverly, MA). The DNA was labelled using direct labelling enzyme (DLE1) and Bionano Prep Direct Label and Stain (DLS) protocol (document number 30206) and then labels sequenced on a Bionano Saphyr instrument.

Total RNA was extracted from five tissues from the focal male (B50597: testes, brain, muscle, liver, and whole body) and four tissues from the female (B40182: ovary, muscle, liver, and whole body) using a QIAGEN RNAeasy Protect kit (cat. no. 74124). RNA quantity was determined using a Qubit 3 fluorometer (Invitrogen Qubit RNA High Sensitivity (HS) Kit (cat. no. Q32852) and RNA integrity (RIN) score determined using an Agilent Fragment Analyzer. RNA paired-end sequencing was performed on an Illumina NovaSeq 6000 machine.

Short read Illumina WGS sequencing of the presumed parents was performed using HMW DNA extracted from kidney tissue from the presumed parents (B40300, B40182) of the focal frog. The library was sequenced with the Illumina NovaSeq 6000 platform with 2×150 bp read length at Psomagen, Inc. (Rockville, MD, USA).

### Genome assembly, curation, evaluation, and annotation

***Assembly***

The assembly was performed using the VGP Pipeline 2.0 with Hi-C phasing ^3^. First, HiFi reads were screened for PacBio adapters using cutadapt 4.0 to remove any matching reads before assembly. Contig assembly was then performed using hifiasm version 0.16.1 in Hi-C phased mode to obtain two phased haplotypes. Contigs of each haplotype were then scaffolded individually using Bionano Solve 3.7.0 followed by YaHS 1.2a.

***Assembly curation***

Haplotype 2 had higher assembly metrics and thus was selected for manual curation and to be used as the main reference. The assembly was decontaminated using the Assembly Screen for Cobionts and Contaminants (ASCC) pipeline ( https://pipelines.tol.sanger.ac.uk/ascc; Aunin *et al.* in prep). Flat files and Hi-C contact maps used in curation were generated via the TreeVal pipeline ^17^. Manual curation was conducted using the rapid curation pipeline documented at ( https://gitlab.com/wtsi-grit/rapid-curation; Wood *et al.* in prep) primarily using PretextView and HiGlass ^18^ with additional insights provided by JBrowse2 ^19^. Scaffolds were visually inspected, and assembly errors corrected as described in Howe *et al.* ^20^. Any identified contamination, missed joins, and mis-joins were amended, and duplicate sequences were tagged and removed.

***Evaluation of the final assembly***

The final assembly was post-processed and evaluated with Nextflow ^21^. DSL2 pipelines “sanger-tol/readmapping” ^22^, “sanger-tol/genomenote” ^23^, and “sanger-tol/blobtoolkit” ^24^. The pipeline sanger-tol/readmapping aligns the Hi-C reads with bwa-mem2 ^25^ and combines the alignment files with SAMtools ^26^. The sanger-tol/genomenote pipeline transforms the Hi-C alignments into a contact map with BEDTools ^27^ and the Cooler tool suite ^28^, which is then visualised with HiGlass ^18^. It also provides statistics about the assembly with the NCBI datasets report ^29^, computes *k*-mer completeness and QV consensus quality values with FastK and MERQURY.FK, and provides a completeness assessment with BUSCO ^30^.

The sanger-tol/blobtoolkit pipeline is a Nextflow port of the previous Snakemake Blobtoolkit pipeline ^31^. It aligns the PacBio reads with SAMtools and minimap2 ^32^ and generates coverage tracks for regions of fixed size. In parallel, it queries the GoaT database ^33^ to identify all matching BUSCO lineages to run BUSCO ^30^. For the three domain-level BUSCO lineages, the pipeline aligns the BUSCO genes to the Uniprot Reference Proteomes database ^34^ with DIAMOND blastp ^35^. The genome was split into chunks according to the density of the BUSCO genes from the closest taxonomically lineage, and each chunk was aligned to the Uniprot Reference Proteomes database with DIAMOND blastx. Genome sequences that had no hit were then chunked with seqtk and aligned to the NT database with blastn ^36^. All outputs were then combined with the blobtools suite into a blobdir for visualisation.

The genome assembly and evaluation pipelines were developed using the nf-core tooling ^37^, using MultiQC ^38^, and making extensive use of the Conda package manager, the Bioconda initiative ^39^, the Biocontainers infrastructure ^40^, and the Docker ^41^ and Singularity ^42^ containerisation solutions.

Only one of the presumed parents, the male frog (B40300), was confirmed to be an actual parent. As a result, we could not utilise parent WGS data for haplotype phasing.

The curated assemblies were submitted to NCBI (BioProject PRJNA928730), and haplotype 2 was annotated by the NCBI Eukaryotic Genome Annotation Pipeline ^43^ using RNA-Seq data from the kidney and liver from the male frog (SAMN32631236) and brain, ovaries, and whole body from the female frog (SAMN39610159). This annotated RefSeq version of this assembly has the accession number GCF_028390025.1 (PRJNA1082331).

Repeats were *de novo* modelled with RepeatModeler (Apptainer v. 1.2.3) ^44^ and then annotated using RepeatMasker (v. 4.1.2-p1) ^45^ with a concatenated library of genome-specific repeats generated from RepeatModeler and the Dfam amphibian repeat library (v. Dfam.h5) ^46^.

## Results

### Genome sequence report

**Figure 1.**
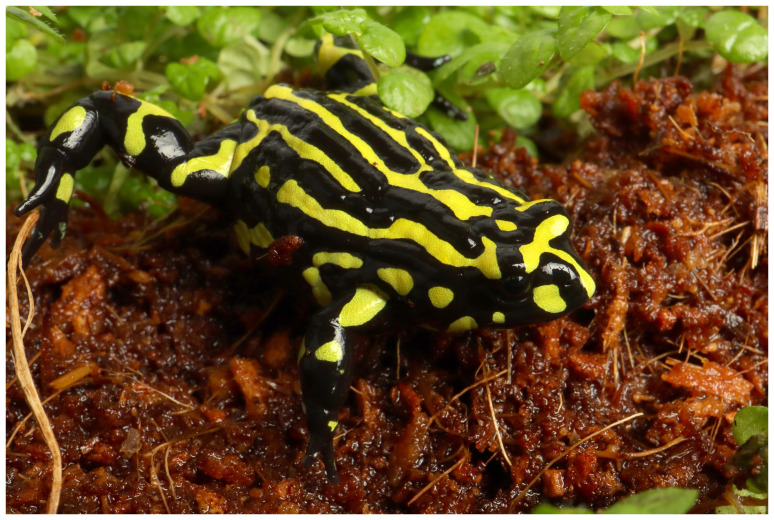
Photograph of a captive-bred *Pseudophryne corroboree*. Photo by C. Doughty.

The assembly of a male *Pseudophryne corroboree* ( Figure 1) resulted in a genome that was 8.87 Gb in length across 12 chromosomes. The genome was sequenced using PacBio HiFi reads, generating a total of 230 Gb from 17,266,474 reads, providing approximately 26.9-fold coverage. Primary assembly contigs were scaffolded with chromosome conformation Hi-C data, which produced 381 Gb from 1,266,207,409 reads, yielding an approximate coverage of 44.5-fold. Specimen and sequencing information are summarised in Table 1.

**Table 1.** Specimen and sequencing data for *Pseudophryne corroboree*.

Project information			
Study title	Pseudophryne corroboree overview		
Umbrella BioProject	PRJNA921726		
Species	*Pseudophryne corroboree*		
BioSample	SAMN32631236		
**NCBI taxonomy ID**	495146		
Specimen information			
**Technology**	**Specimen ID (sex)**	**BioSample accession**	**Organism part**
PacBio long read sequencing	B50597 (male)	SAMN32631236	Kidney, liver
Hi-C sequencing	B50597 (male)	SAMN32631236	Liver
RNA sequencing	B50597 (male)	SAMN32631236	Brain, muscle, testes, liver, whole body
RNA sequencing	B40182 (female)	SAMN39610159	Ovary, muscle, liver, whole body
Illumina DNA sequencing	B40182 (female)	SAMN39610159	Kidney
Illumina DNA sequencing	B40300 (male)	SAMN40858189	Kidney

**Figure 2.**
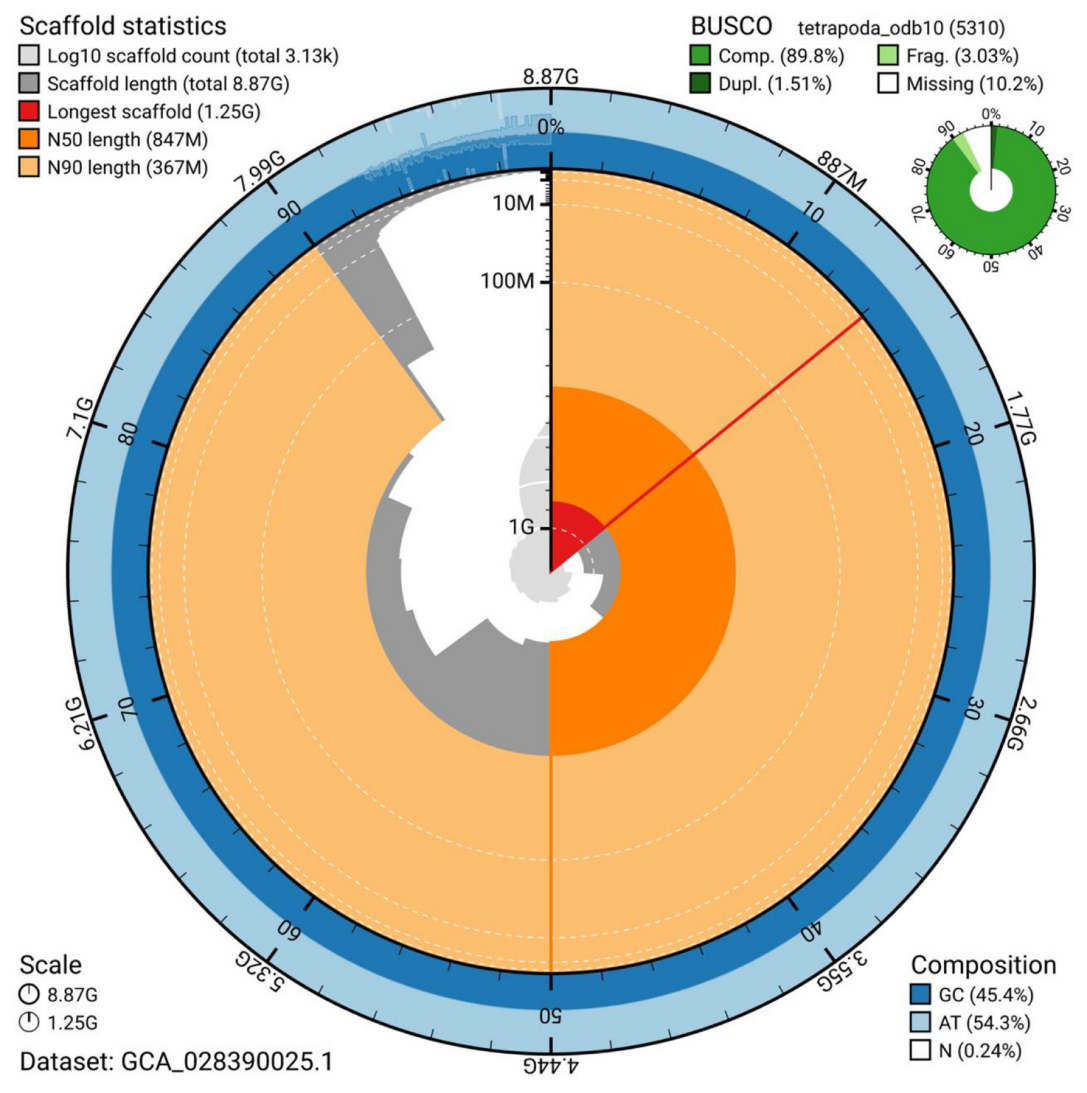
Genome assembly metrics for *Pseudophryne corroboree*, aPseCor3.hap2. The BlobToolKit snail plot shows N50 scaffold metrics and BUSCO gene completeness. An interactive version of this figure is available at: https://blobtoolkit.genomehubs.org/view/GCA_028390025.1/dataset/GCA_028390025.1/snail.

**Figure 3.**
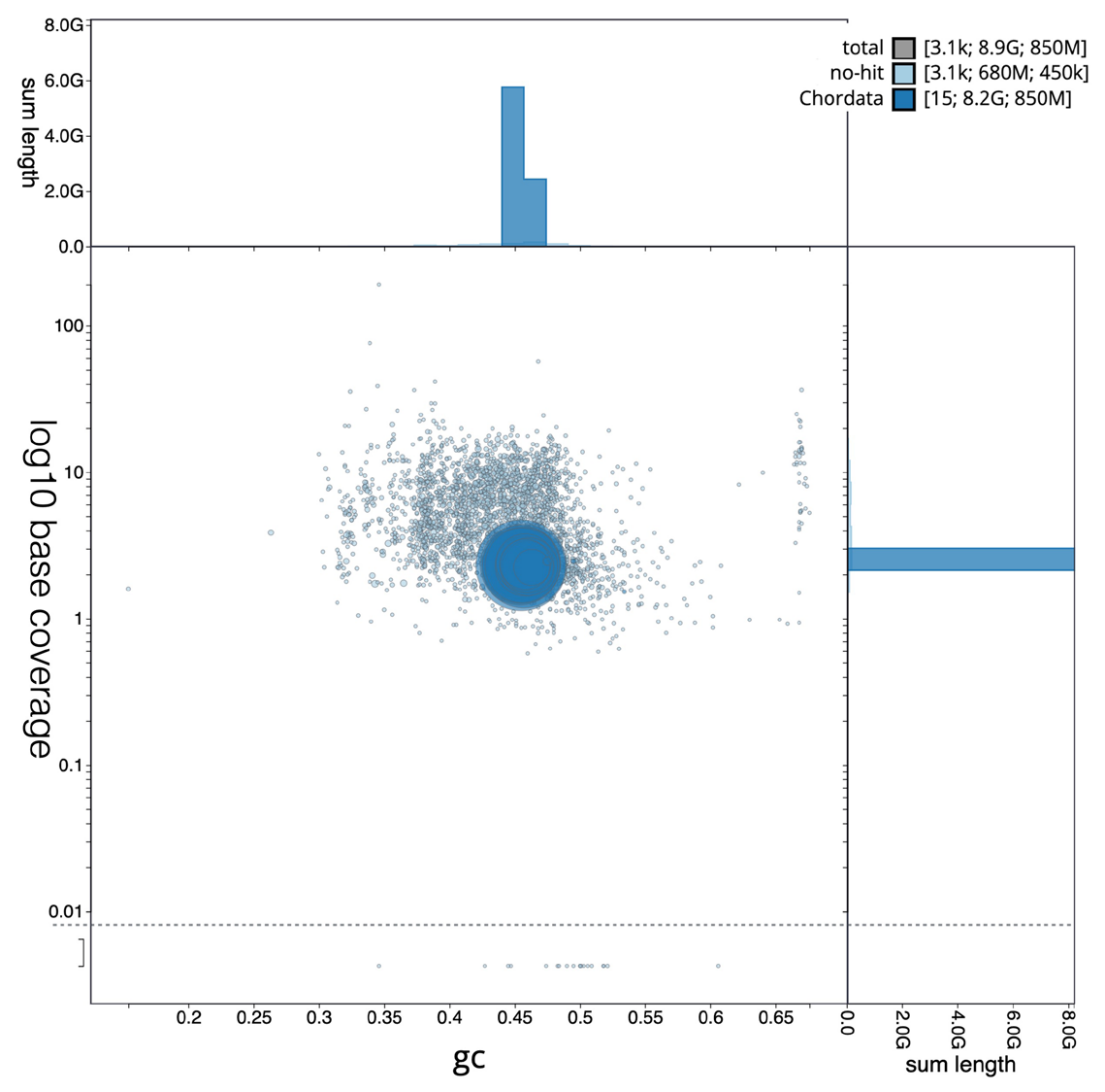
BlobToolKit plot of base against GC proportion for genome assembly *Pseudophryne corroboree*, aPseCor3.hap2. Circles are sized in proportion to sequence length. Histograms show the distribution of sequence length sum along each axis. Base coverage values are log10 transformed. An interactive version of this figure is available at: https://blobtoolkit.genomehubs.org/view/GCA_028390025.1/dataset/GCA_028390025.1/blob?plotShape=circle.

**Figure 4.**
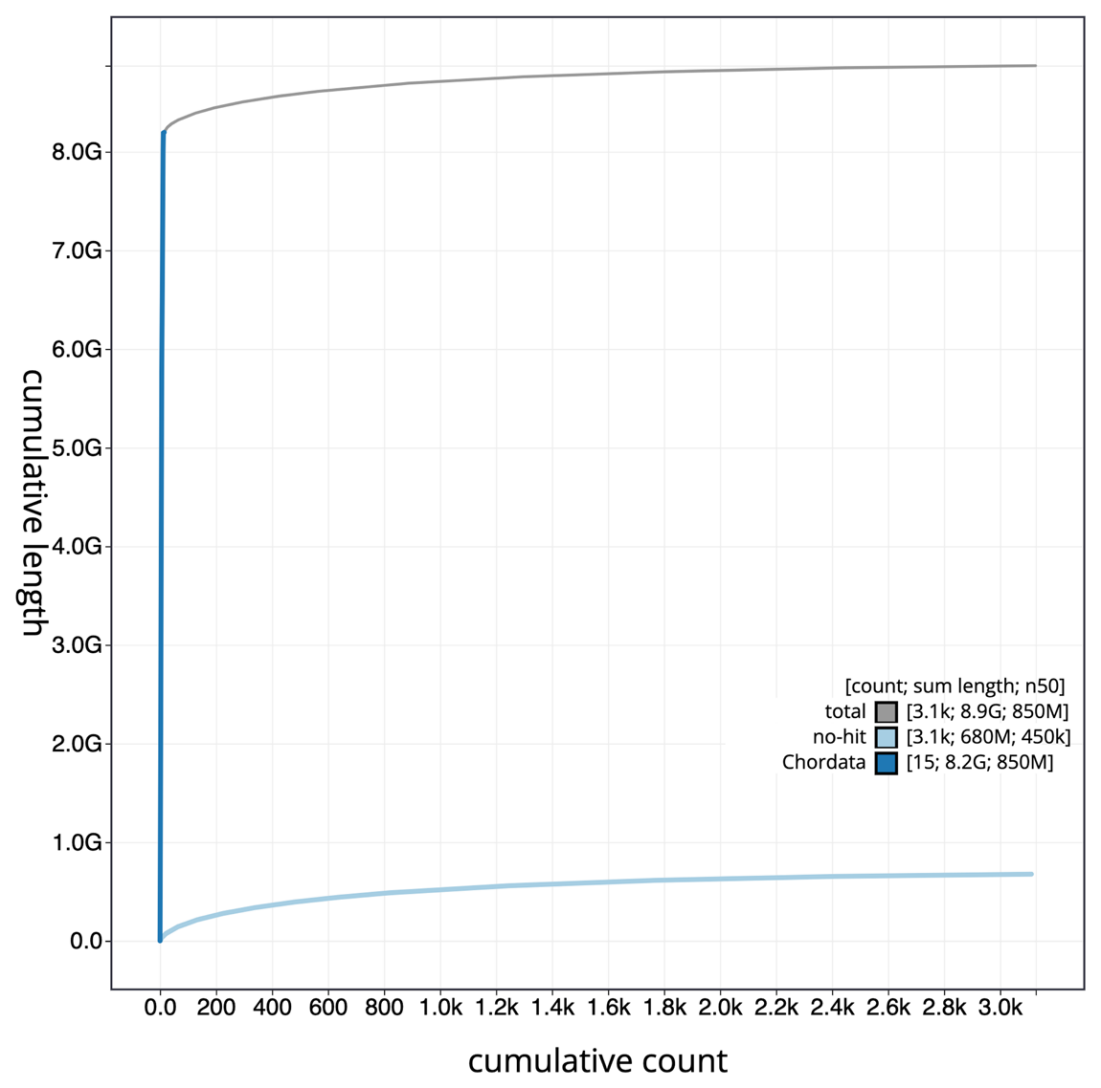
Genome assembly of *Pseudophryne corroboree*, aPseCor3.hap2: BlobToolKit cumulative sequence plot. The grey line shows cumulative length for all sequences. Coloured lines show cumulative lengths of sequences assigned to each phylum using the buscogenes taxrule. An interactive version of this figure is available at: https://blobtoolkit.genomehubs.org/view/GCA_028390025.1/dataset/GCA_028390025.1/cumulative.

**Figure 5.**
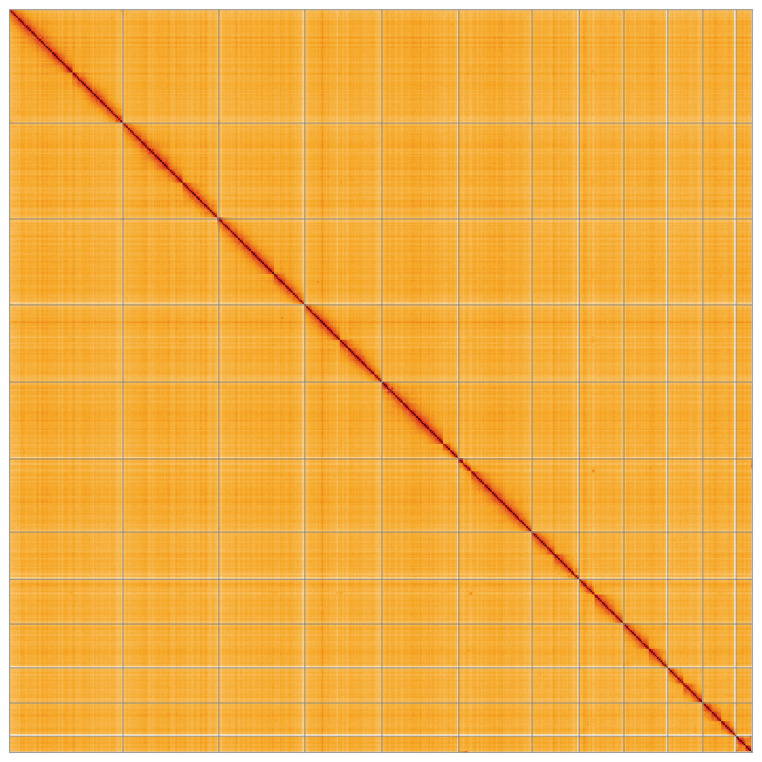
Hi-C contact map of the curated aPseCor3.hap2 assembly, visualised using HiGlass. Chromosomes are shown in order of size from left to right and top to bottom. An interactive version of this figure may be viewed at https://genome-note-higlass.tol.sanger.ac.uk/l/?d=RTD6MMzAT-mgaBhXdbrKig.

Manual assembly curation corrected 280 missed joins or mis-joins and 1 haplotypic duplication, reducing the assembly length by 0.79% and the scaffold number by 11%, and increasing the scaffold N50 by 24.6%. The final assembly has a total of 3,127 scaffolds, with 2,699 gaps, and a large scaffold N50 of 846.9 Mb and a contig N50 of 6.8 Mb ( Table 2), well surpassing the VGP minimum metrics of 10 and 1 Mb, respectively ^2^. The snail plot in Figure 2 provides a summary of the assembly statistics, while the distribution of assembly scaffolds by GC proportion and coverage is shown in Figure 3. The cumulative assembly plot in Figure 4 shows curves for subsets of scaffolds assigned to different phyla. Most (92.3%) of the assembly sequence was assigned to 12 chromosomal-level scaffolds. These chromosome-scale scaffolds were confirmed by the Hi-C data and are named in order of size ( Figure 5; Table 3).

**Table 2.** Genome assembly data for *Pseudophryne corroboree*, aPseCor3.hap2.

Genome assembly		
Assembly name	aPseCor3.hap2	
Assembly accession haplotype	GCA_028390025.1 haplotype 2; GCA_028390055.1 haplotype 1	
Assembly length	8872758793	
Chromosome assembly length	8190274449	
Number of contigs	5826	
Contig N50 length (Mb)	6.8	
Number of scaffolds	3127	
Scaffold N50 length (Mb)	846.9	
Longest scaffold (Mb)	947.25	
Assembly metrics *		*Benchmark*
Consensus quality (QV)	58.5	*≥ 50*
*k*-mer completeness	97.66%	*≥ 95%*
BUSCO **	C:89.8% [S:88.3%, D:1.5%], F:3.0%, M:7.2%, n:5310	*C ≥ 95%*
Percentage of assembly mapped to chromosomes (N=12)	92.3%	*≥ 95%*
Genome annotation of assembly GCF_028390025.1-RS_2024_02 at RefSeq		
Number of protein-coding genes	24591	
Number of non-coding genes	128683	
Number of gene transcripts	183540	
Genome repeat content		
**Repeat element**	**Number of elements**	**% of genome**
DNA transposons	1749727	11.7
LINEs	821317	5.75
SINEs	134337	1.04
LTRs	1435160	27.15
Simple	1353528	0.82
Unclassified	10152528	28.08

* Assembly metric benchmarks are adapted from column VGP-2020 of “Table 1: Proposed standards and metrics for defining genome assembly quality” from Rhie *et al.* (2021). ** BUSCO scores based on the tetrapoda_odb10 BUSCO reference set using version 5.4.3. C = complete [S = single copy, D = duplicated], F = fragmented, M = missing, n = number of orthologues in comparison. A full set of BUSCO scores is available at https://blobtoolkit.genomehubs.org/view/GCA_028390025.1/dataset/GCA_028390025.1/busco.

**Table 3.** Chromosomal pseudomolecules in the genome assembly of *Pseudophryne corroboree*.

GenBank accession	Name	Length (Mb)	GC%
CM051801.1	1	1251.52	45.5
CM051802.1	2	1055.99	46
CM051803.1	3	808.54	46
CM051804.1	4	947.25	45.5
CM051805.1	5	852.35	45.5
CM051806.1	6	846.89	45.5
CM051807.1	7	518.98	46
CM051808.1	8	493.02	46
CM051809.1	9	483.89	46
CM051810.1	10	387.42	46
CM051811.1	11	366.57	46
CM051812.1	12	177.84	46.5

The estimated Quality Value (QV) of the final assembly is 58.5 (no more than 1 error per ~1Mb) and BUSCO v5.5.0 completeness of 89.8% (single = 88.3%, duplicated = 1.5%), using the tetrapoda_odb10 reference set ( *n* = 5,310). *K-mer* completeness for the combined haplotypes was 97.66% complete (aPseCor3.hap1 = 91.91% and aPseCor3.hap2 = 91.96%).

A considerable portion (76%) of the *P. corroboree* genome consisted of repeats, with the majority of these (27%) classified as Long Terminal Repeats (LTRs; Table 2).

## Ethics and consent

Frogs were humanely euthanised following University of Melbourne (Victoria, Australia) Animal Ethics permit #10267. Tissue samples were exported for sequencing at Rockefeller University (New York, USA) under Australian wildlife trade export permit (#PWS2020-AU-001530).

## Data Availability

NCBI Archive: *Pseudophryne corroboree* overview. The genome sequence is released openly for reuse. The *Pseudophryne corroboree* genome sequencing initiative is part of the Vertebrate Genomes Project (VGP). Further, raw data and assembly accession identifiers are reported in Table 1 and Table 2. Relevant software tool versions and sources are listed in Table 4. Interactive genome figures are available at https://blobtoolkit.genomehubs.org/.
