# Supplementary material for: A chromosome-level reference genome for the critically endangered Southern Corroboree frog ( Pseudophryne corroboree)
Source: Wellcome Open Res. 2025 Apr 30;10:228. [Version 1] doi: 10.12688/wellcomeopenres.23820.1 (PMC12314482; doi:10.12688/wellcomeopenres.23820.1)
Supplement: Peer review history

# Reviewer report: Reviewer response for version 1

**DOI:** 10.21956/wellcomeopenres.26272.r126531

**Published:** 2025-07-31

**Authors:** Richard Edwards

**Affiliations:** 1 School of Biotechnology and Biomolecular Sciences, University of New South Wales, Sydney, NSW, Australia; 2 Oceans Institute, University of Western Australia, Perth, WA, Australia

This is another high-quality genome assembly, sequenced as part of the VGP. The overall quality of the assembly looks good, especially for a genome so big. 

 The only slight concern is the rather low BUSCO completeness, combined with a large number of unplaced contig/scaffolds that BlobToolkit cannot assign as Chordata. This is a bit hard to judge in a large, repetitive amphibian genome, as the BUSCO completeness could be due to a poorly representative OrthoDB dataset, whilst the unplaced unassigned sequences could be largely repetitive. The manuscript presents the data as-is, so this is not a problem, but it would be useful if some additional analysis could confirm whether (1) the unplaced sequences appear to be mostly repetitive, and (2) the Missing BUSCO genes tend to be missing from other frog/toad genomes.

 On a related note, it would be useful to know what the expected genome size and karyotype is for this species. Relating the summed chromosome size to expected genome size is helpful for interpreting the importance of the unplaced sequences. Is the expected karyotype 2 *n*=24?

 It is a pity that the parental phasing was not possible. Can some additional details be provided regarding why parentage was presumed and how actual parentage was determined?

 Finally, it would be good to have information about sex chromosomes if known. Is this a ZW species? If so, it might be possible to propose which is the sex chromosome using BUSCO content and other frog/toad genomes.

Are sufficient details of methods and materials provided to allow replication by others?

Yes

Is the rationale for creating the dataset(s) clearly described?

Yes

Are the datasets clearly presented in a useable and accessible format?

Yes

Are the protocols appropriate and is the work technically sound?

Yes

Reviewer Expertise:

Genome assembly/curation, biodiversity genomics, bioinformatics

I confirm that I have read this submission and believe that I have an appropriate level of expertise to confirm that it is of an acceptable scientific standard.

# Reviewer report: Reviewer response for version 1

**DOI:** 10.21956/wellcomeopenres.26272.r123629

**Published:** 2025-06-06

**Authors:** Yanbo Sun

**Affiliations:** 1 Yunnan University, Kunming, China

The study provides a technically robust and well-motivated chromosome-level genome assembly for P. corroboree. To enhance reproducibility and accessibility:

 Table Enhancement: Include SRA accessions for all sequencing datasets (e.g., Hi-C: SAXXX; RNA-Seq: SAXXX).

 Supplemental Material: Share Snakemake/Nextflow workflow scripts via GitHub or Zenodo.

 Annotation Methods: Briefly describe evidence sources (e.g., ab initio prediction, transcript alignment).

 Synteny or collinearity studies with closer relatives (e.g., Pseudophryne pengilleyi) are needed

Are sufficient details of methods and materials provided to allow replication by others?

Partly

Is the rationale for creating the dataset(s) clearly described?

Yes

Are the datasets clearly presented in a useable and accessible format?

Yes

Are the protocols appropriate and is the work technically sound?

Yes

Reviewer Expertise:

Evolutionary genomics of amphibians and reptiles

I confirm that I have read this submission and believe that I have an appropriate level of expertise to confirm that it is of an acceptable scientific standard.
